# Loading with Biomolecules Modulates the Antioxidant Activity of Cerium-Doped Bioactive Glasses

**DOI:** 10.1021/acsbiomaterials.2c00283

**Published:** 2022-06-13

**Authors:** Gigliola Lusvardi, Francesca Fraulini, Sergio D’Addato, Alfonso Zambon

**Affiliations:** † Department of Chemical and Geological Sciences, University of Modena and Reggio Emilia, Via G.Campi 103, Modena 41125, Italy; ‡ Department of Physical, Information and Mathematical Sciences, University of Modena and Reggio Emilia, Via G. Campi 213/a, Modena 41125, Italy; § Istituto Nanoscienze−CNR, Via G. Campi 213/a, Modena 41125, Italy

**Keywords:** bioactive glasses, cerium, biomolecules, antioxidant activities

## Abstract

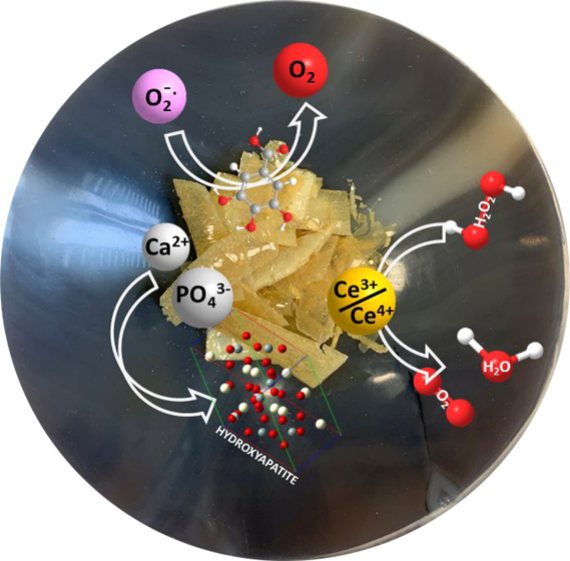

In order to identify new bioactive glasses (BGs) with optimal antioxidant properties, we carried out an evaluation of a series of cerium-doped BGs [Ce-BGs—H, K, and mesoporous bioactive glasses (MBGs)] loaded with different biomolecules, namely, gallic acid, polyphenols (POLY), and anthocyanins. Quantification of loading at variable times highlighted POLY on MBGs as the system with the highest loading. The ability to dismutate hydrogen peroxide (catalase-like activity) of the BGs evaluated is strongly correlated with cerium doping, while it is marginally decreased compared to the parent BG upon loading with biomolecules. Conversely, unloaded Ce-BGs show only a marginal ability to dismutate the superoxide anion (SOD)-like activity, while upon loading with biomolecules, POLY in particular, the SOD-like activity is greatly enhanced for these materials. Doping with cerium and loading with biomolecules give complementary antioxidant properties to the BGs investigated; combined with the persistent bioactivity, this makes these materials prime candidates for upcoming studies on biological systems.

## 1 Introduction

Bioactive glasses (BGs) find application in medicine as bone fillers, scaffolds, and implant coatings due to their ability to stimulate bone regeneration;^1,2^ since the development of the first BG (45S5 Bioglass),^3^ their therapeutic use has expanded significantly, supporting and/or generating many biomedical applications. BGs are particularly versatile materials, and a variation of their forms (powders, coatings, 3D-scaffolds, and fibers) and compositions (addition of therapeutic inorganic ions, TIIs) have been shown to improve a range of relevant properties such as osteogenesis, angiogenesis, antibacterial activity, and cementogenesis.^4,5^

Our research group has investigated the effect of the addition of TIIs to BGs,^4,6−9^ with a focus on the antioxidant properties and therapeutic applications of cerium-doped BGs (Ce-BGs).^5,10−16^ To elucidate the redox behavior (Ce^3+^/Ce^4+^ ratio) of Ce-BGs,^17−19^ we performed studies with X-ray photoelectron spectroscopy (XPS) and near-edge X-ray absorption spectroscopy; the same study was also carried out on Ce-BGs after enzymatic-like or enzyme-mimetic tests were performed with the aim of accurately assessing the evolution of the Ce^3+^/Ce^4+^ ratio.^20,21^ Alginate-treated Ce-BGs were also studied to verify the maintenance of the Ce^3+^/Ce^4+^ ratio.^22^ Ce-BGs present antioxidant, antibacterial, osteogenic, and angiogenic properties and are promising therapeutic options for wound healing and tissue repair.^23,24^

Crucially, the insertion of a biomaterial performed through surgery is often followed by tissue damage and inflammation;^25−27^ correlated production of reactive oxygen species (ROS) induces a condition of oxidative stress, which in turn enhances inflammation, causing further generation of ROS. Due to this feedback, post-surgery inflammation could take a long time to achieve complete recovery, thus the ability to convert ROS to non-dangerous species is a desirable feature of a biomaterial.^25,28^ The antioxidant properties of BGs are strictly correlated to their composition and reactivity; studies performed on 45S5 doped with fluorine reveal an increase of lipid peroxidation, ROS production in MG-63 osteoblast cells, inhibition of the pentose phosphate pathway, glucose 6-phosphate dehydrogenase activity, and glutathione activity.^29,30^ Similarly, the introduction of copper into 45S5 increases the ROS production in human osteosarcoma cells (HOS).^29,30^

We have demonstrated that Ce-BGs have antioxidant activities mimicking the catalase (CAT) and superoxide dismutase (SOD) enzymes and are thus promising materials to limit the level of ROS upon implanting.^17−19,21,24,31^

Recently, considerable attention has been paid to biomaterials loading with biomolecules, which appears to be a promising strategy to target specific cellular signals in the relevant tissue directly from the biomaterial’s surface; biomolecules of natural origin are of particular interest in this field for their antioxidant, anti-tumor, antibacterial, anti-inflammatory, vasoprotective, and bone-stimulating actions.^32−38^ Among the biomolecules investigated, several studies use antioxidants such as gallic acid (GA), POLY pure, and combined with organic (chitosan) and inorganic (Mg/Al-layered double hydroxide, NPs) carriers to take advantage of their antioxidant, antibacterial, and antitumor properties.^39−41^ Surface loading is also applicable to BGs, whose surface reactivity is important for promoting interfacial bond formation with the host tissue and at the same time presents reactive hydroxyl groups that favor the loading of biomolecules.^42,43^ This strategy allows the slow release of biomolecules and promotes their bioavailability; thus, loaded BGs are used not only for tissue regeneration but also for other settings such as, for example, soft tissue and wound healing.^4,44,45^

Here, we report the multiparametric evaluation of a series of Ce-BGs [H, K, and mesoporous bioactive glasses (MBG)] loaded with different biomolecules, namely, GA, POLY, and anthocyanins (ANTO), as a function of their loading extent, stability of the BGs upon loading, antioxidant properties, and bioactivity.^53,54^

## 2 Experimental Section

### 2.1 BG Preparation

Twelve BGs (Table 1) containing different amounts of cerium (0, 1.2, 3.6, and 5.3 mol %) were synthetized by traditional melt quenching (H and K series) and sol–gel EISA-modified methods (MBG series) as previously described.^18,22,24,46^ The BGs were ground to two different sizes, namely fine and coarse: for H and K series, fine are <180 μm and coarse are >180 and <355 μm; for MBG series, fine are <250 μm and coarse are >250 μm.

**Table 1:** Nominal Composition (mol %) of Ce-BGs

**BG**	**SiO_2_**	**Na_2_O**	**CaO**	**P_2_O_5_**	**CeO_2_**
**H0**	46.2	24.3	26.9	2.6	
**H1.2**	45.6	24.0	26.6	2.6	1.2
**H3.6**	44.5	23.4	26.0	2.5	3.6
**H5.3**	43.4	23.2	25.7	2.4	5.3
**K0**	50	25	25		
**K1.2**	49.4	24.7	24.7		1.2
**K3.6**	48.2	24.1	24.1		3.6
**K5.3**	47.3	23.7	23.7		5.3
**MBG0**	80		15	5	
**MBG1.2**	79.1		15.0	4.9	1.2
**MBG3.6**	77.1		14.5	4.8	3.6
**MBG5.3**	75.8		14.2	4.7	5.3

### 2.2 Surface Activation

Surface activation of each BG was carried out according to the literature^39,40,47^ in order to free the hydroxyl groups on the BG surface and promote loading. Briefly, 0.4 g of each BG were suspended in 5 mL of acetone and washed for 5 min in an ultrasonic bath, then rinsed three times with 5 mL of double distilled water, under sonication, and finally air-dried at room temperature overnight.

### 2.3 Loading with Biomolecules

BGs were loaded with GA, POLY, and ANTO, see Table 1for their compositions. GA was purchased from Riedel de Haen. Mixtures POLY and ANTO were generous donations by Proff Lorenzo Tassi and Laura Pigani of the Department of Chemistry and Geological Sciences, University of Modena and Reggio Emilia, respectively. POLY were extracted from chestnut flour and contained 46 wt % of GA.^48^ ANTO was derived from a powdered extract of red grape skins, and their composition is as follows: ANTO (15 wt %), tannins (55 wt %), other POLY, organic acids, and impurities (30 wt %). ANTO mainly consist of malvidin (32 wt %), delphinidin (14 wt %), petunidin (12 wt %), peonidin (7 wt %), and cyanidin (3 wt %).^49^ 1.0 mg/mL loading solutions were prepared by dissolving the biomolecules in double distilled water for 2 h under magnetic stirring. The loading was carried out by soaking 0.1 g of each BG for 3 or 6 h at 37 °C in 5 mL of biomolecules’ loading solution. All the holders were covered with aluminum foils in order to prevent light irradiation.

#### 2.3.1 UV–Vis Analyses—Folin and Ciocalteau Method—Gallic Acid Equivalent Determination

A modified Folin and Ciocalteau (F&C) method^39,40^ was utilized to quantify the amount of biomolecules loaded onto the BG directly on the solid mixture. The results are reported as the percentage of gallic acid equivalents (GAE), the most common spectrophotometric parameter for the estimation of antioxidant properties. 0.1 g of grafted BG were mixed with 8 mL of double distilled water, 0.5 mL of F&C reagent (Sigma-Aldrich), and 1.5 mL of 20% (*p*/*V*) Na_2_CO_3_ (Sigma-Aldrich) solution. After 2 h, UV–vis measurements were carried out on the resulting solution by means of a UV–vis spectrophotometer (JASCO V-570). A calibration curve was prepared with eight solutions at defined GA concentrations (0.0015, 0.0030, 0.0060, 0.0090, 0.0150, 0.0300, 0.0600, and 0.3000 mg/mL). The results are reported as GAE % in weight.^41,50,51^

#### 2.3.2 Elemental Analysis

Elemental analysis (EA) was carried out with a FLASH 2000 Thermo Fisher analyzer in order to quantify the biomolecules in the loaded BGs by the measurement of % C. These results were then compared with those obtained with the F&C method; it must be noted that this comparison holds only qualitative value for POLY and ANTO which are complex mixtures of several biomolecules of variable molecular weight.

#### 2.3.3 Specific Surface Area Determination

The specific surface area (SSA) was evaluated before and after loading in order to assess possible textural changes arising from this process. SSA was determined by nitrogen adsorption porosimetry using a Micromeritics Chemisorb 2750 and the Brunauer–Emmett–Teller (BET) method.^52^

#### 2.3.4 Fourier Transform Infrared Spectroscopy

Fourier transform infrared (FTIR) spectra were collected on a PerkinElmer 1600 spectrometer in the range 400–4000 cm^–1^ to verify the presence of biomolecules loaded on the BGs; we focused on the characteristic bands of GA.

#### 2.3.5 X-ray Photoelectron Spectroscopy

X-ray photoelectron spectroscopy (XPS) was used to obtain information on the relative amount of Ce^3+^ and Ce^4+^ on the surface of the samples. XPS spectra were collected at normal emission using a hemispherical electron analyzer and Mg Kα photons as the exciting probe. Because of the insulating nature of the samples, the XPS spectra were affected by charging effects, which resulted in a shift of the binding energy of the photoemission peaks. The shift ranges between 4.6 and 5.5 eV in different samples. In spite of the low concentration of Ce present on the surface of the investigated samples, we managed to measure Ce 3d XPS spectra with an acceptable signal-to-noise ratio using long acquisition times (approximately 4 h for the Ce 3d spectrum). Given the limited probing depth of the XPS technique, the information obtained can be related only to the first few nanometers below the surface of the investigated samples.

The techniques described in Sections 2.3.3, 2.3.4, and 2.3.5 have been performed on the most promising BGs, mainly MBGs because of their higher loading values.

### 2.4 Release of Ions from BGs in the Loading Solutions

The amount of the most relevant ions (silicon, calcium, and cerium) released from BGs in the loading solutions was measured in order to estimate the extent of BG dissolution upon loading. The amounts of silicon and calcium were analyzed by an ICP-OES Optima 4200 DV PerkinElmer spectrometer and that of cerium by an ICP–MS HR-MS-XSeries II Thermo Fisher mass spectrometer.

The amounts of silicon, calcium, phosphorus, and cerium were also evaluated with the same methods in a simulated body fluid (SBF) used for the in vitro bioactivity assessment (Section 2.6) but only for the most promising BGs.

### 2.5 Antioxidant Activity Assays

The antioxidant properties of the loaded BGs were estimated by enzymatic assays as their ability to remove the H_2_O_2_ radical and superoxide anion O_2_^•–^, two of the most significant ROS species. In analogy with the role of enzymes catalase and SOD, these were named CAT-like activity and SOD-like activity, respectively.

#### 2.5.1 CAT-like Activity

CAT-like activity tests were performed using an Amplex-Red kit (Thermo Fisher, Cat N A22188) with a TECAN GeniosPro microplate reader. In this assay, the presence of H_2_O_2_ is detected by its reaction in a 1:1 stoichiometry with an Amplex Red reagent catalyzed by peroxidase to produce a red-fluorescent oxidation product, resorufin. CAT-like activity is defined as the percentage of H_2_O_2_ decomposed at the end of the assay.

#### 2.5.2 SOD-like Activity

SOD-like activity tests were performed using the SOD determination kit (Sigma-Aldrich) adapted for a UV–vis spectrophotometer (JASCO V-570). The principle was also illustrated in previous published results.^19^ In this assay, the SOD-like activity is expressed as the inhibition rate (I.R. %) of the formation of a water-soluble formazan dye formed upon reduction of a tetrazolium salt, WST-1, by the superoxide anion catalyzed by xanthine oxidase and inhibited by SOD.^19^

### 2.6 *In vitro* Bioactivity Assessment

The loaded BGs were soaked in SBF at 37 °C for 24, 96, and 168 h^55^ in order to verify the retention of bioactivity [formation of an apatite layer, hydroxyapatite, Ca_10_(PO_4_)_6_(OH)_2,_ HA] by the loaded materials. After soaking, mineralogical evaluations were carried out using an X’Pert PRO-PANAnalytical diffractometer in order to detect the formation of HA.^55^

## Results and Discussion

### 3.1 GAE % Determination

As discussed in the Experimental Section, the extent of loading on each BG was evaluated by the F&C method^39,40^ and EA. All BGs were analyzed by EA before loading to rule out any carbonation of the unloaded (UL) BG, which would influence the % C of the final material. Whenever possible, we compared our results with literature data on similar systems.

#### 3.1.1 Loading with GA

**Figure 1:**
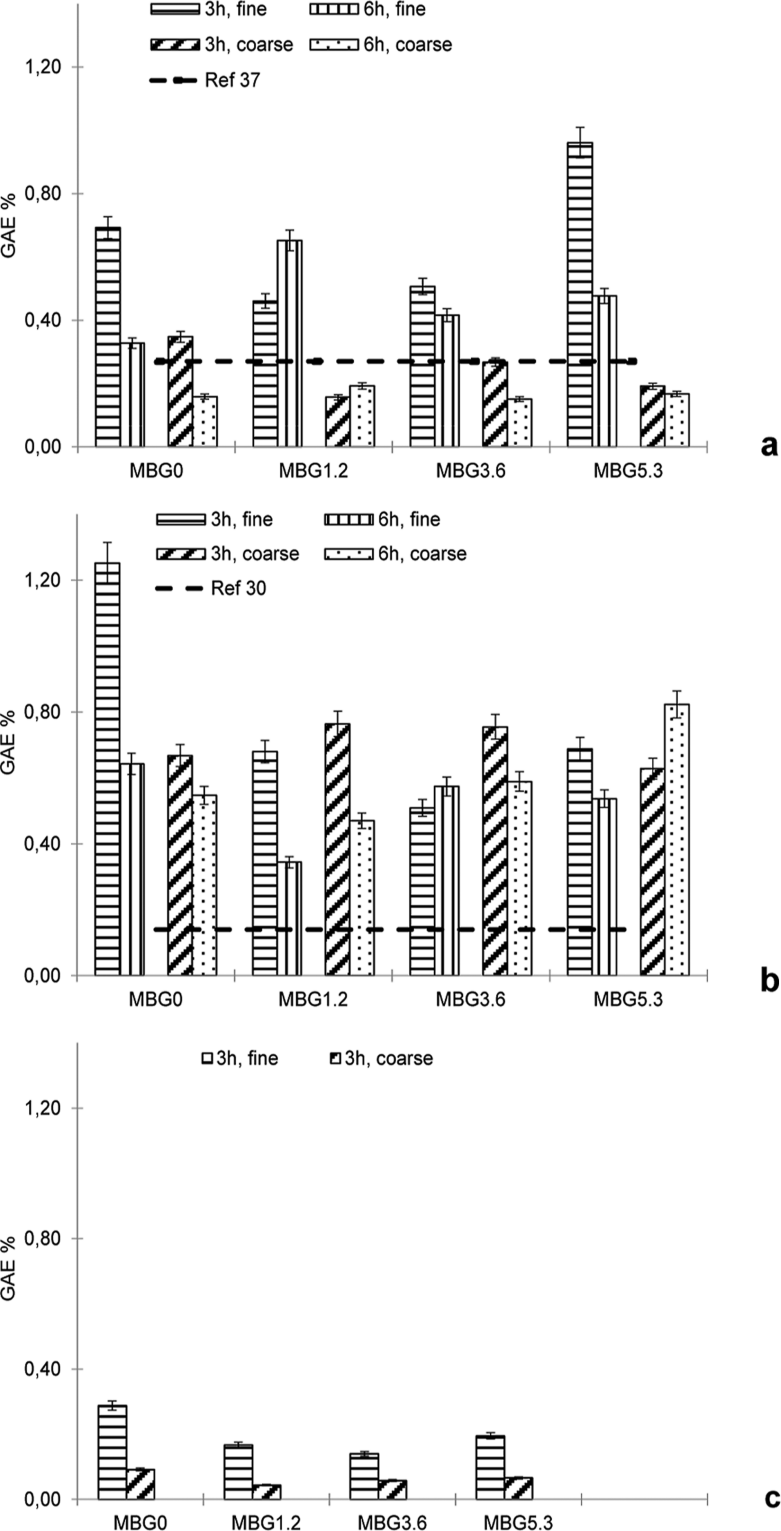
GAE % for MBGs loaded with GA (a), POLY (b), and ANTO (c). Reference literature values from refs (37) and^30^ are reported as dotted lines.

H and K BGs show a similar value of 0.20 GAE % regardless of the cerium content, size, and loading time (Table 1, Supporting Information). Literature data report a 0.27 GAE % for phosphosilicate glasses obtained by the traditional melting method and loaded with GA.^39^Figure 1a shows the results obtained from loading of MBGs with GA as a function of time and size. As to be expected by their higher surface area, the loading values measured for MBGs are larger than those of both literature and H and K BGs. The cerium-free MBG after 3 h of loading shows 0.70 and 0.40 GAE % for fine and coarse size, respectively. Cerium does not seem to significantly affect the extent of loading, yielding, for example, GAE % values in the range of 0.65–0.95 for all compositions for fine size after 3 h. A significant reduction of loading is observed at the coarse size, with values in the range 0.15–0.35 GAE %. Interestingly, for both sizes, the loading results at 3 h are generally in line with or better than those at 6 h.

#### 3.1.2 Loading with POLY

Literature data for phosphosilicate glasses loaded with natural POLY reports 0.14 and 0.08 GAE % for green tea leaves and red grape skin-derived POLY, respectively.^32^ We measured a comparable loading extent for our chestnut-derived POLY, with maximum values ∼0.30 GAE % for both H and K BGs (Table 1, Supporting Information). Analogously with what was observed for GA, MBGs show a higher loading than H and K BGs: after 3 h, cerium-free MBGs present ∼1.20 and 0.60 GAE % for fine and coarse sizes, respectively (Figure 1b), and the loading time does not significantly affect the GAE %. Again, in line with what was observed for GA loading, the amount of cerium does not significantly affect the loading of POLY. Interestingly, at 3 h, POLY GAE % values fall in the 0.60–1.20% range, higher than those obtained with GA (Figure 1a vs Figure 1b), suggesting a higher affinity of POLY for the MBGs.

#### 3.1.3 Loading with ANTO

For this loading, we had no literature data to compare. Nevertheless, the values were significantly lower than those obtained by the loading with GA and POLY (Table 1, Supporting Information). For H and K BGs, we obtained GAE % < 0.10 for all sizes, cerium amount and loading times. For MBGs, we observed a maximum value of ∼0.30 and 0.10% for fine and coarse sizes, respectively (Figure 1c). These results suggest a lower affinity of the ANTO mixture for the BGs.

#### 3.1.4 Elemental Analysis

Results of EA are in line with those obtained by the F&C method, confirming the extent of loading (Table 1, Supporting Information). Table 2 summarizes the loading values (expressed as GAE %) of Ce-MBGs obtained by EA and the F&C method.

**Table 2:** Loading of Biomolecules on MBGs, Expressed as GAE %

		**GA**				**POLY**				**ANTO**	
		**3 h**		**6 h**		**3 h**		**6 h**		**3 h**	
**size**	**BGs**	**EA**	**F&C**	**EA**	**F&C**	**EA**	**F&C**	**EA**	**F&C**	**EA**	**F&C**
**coarse**	**MBG0**	0.10	0.35	0.28	0.16	0.62	0.67	0.28	0.55	N.A.^a^	0,09
	**MBG1.2**	0.14	0.16	0.06	0.19	1.24	0.76	0.30	0.47	N.A.^a^	0,04
	**MBG3.6**	0.12	0.27	0.00	0.15	1.04	0.76	0.06	0.59	N.A.^a^	0,06
	**MBG5.3**	0.48	0.19	0.80	0.17	1.58	0.63	0.46	0.82	N.A.^a^	0,07
**fine**	**MBG0**	1.02	0.69	0.28	0.33	0.96	1.25	0.46	0.64	0.28	0,29
	**MBG1.2**	0.66	0.46	0.66	0.65	1.34	0.68	0.84	0.34	0.26	0,17
	**MBG3.6**	1.20	0.51	0.72	0.42	0.52	0.51	1.42	0.57	0.32	0,14
	**MBG5.3**	1.46	0.96	0.86	0.48	1.10	0.69	1.08	0.54	0.36	0,20

a Not assessed (N.A.).

#### 3.1.5 Selection of Biomolecules and Loading Times

Given their lower loading content, optimization of ANTO BGs was not pursued any further in this study. However, the selected ANTO BG samples were used for comparison in the evaluation of the antioxidant properties of the loaded BGs.

Similarly, for all the BGs and biomolecules assessed, the loading results at 3 h are generally in line with or better than those at 6 h, suggesting that the loading equilibrium is reached within this timeframe. We thus deemed the 3 h loading time preferable for the optimized materials, in order to limit BG dissolution upon loading.

### 3.2 Estimation of BG Dissolution upon Loading

**Figure 2:**
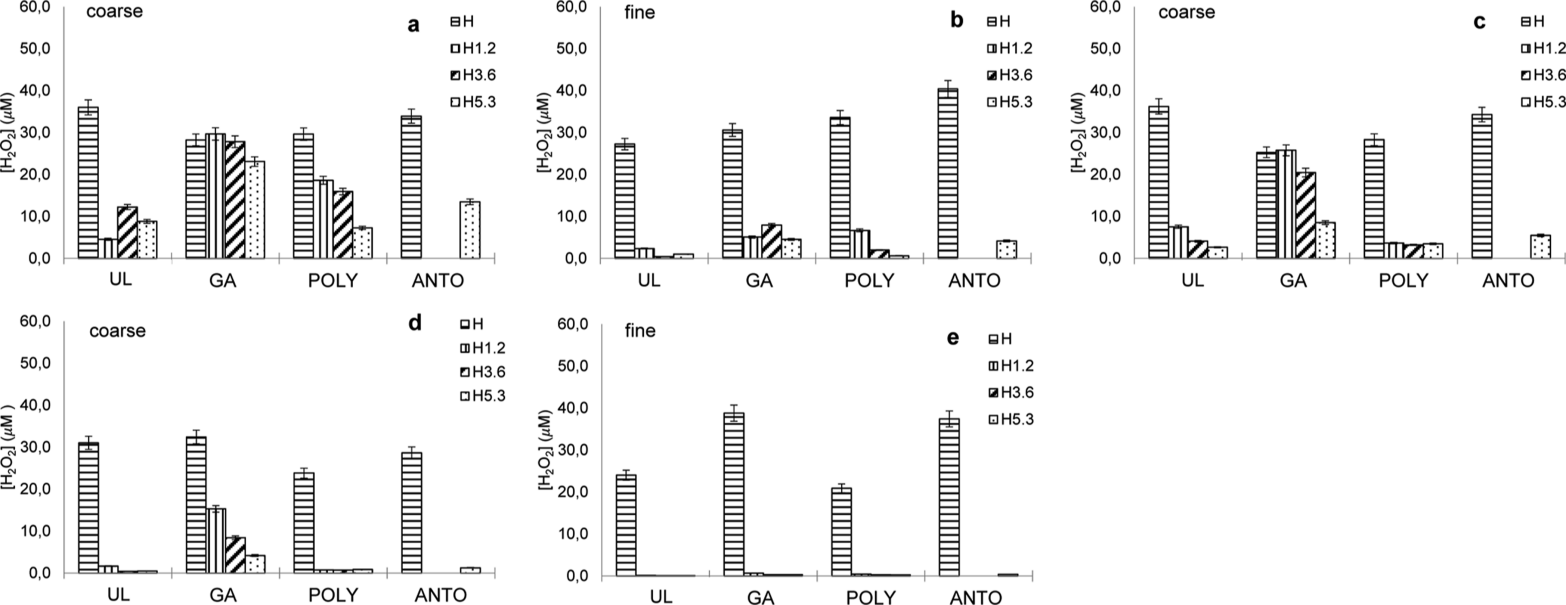
Residual H_2_O_2_ concentration (μM) of a 50 μM H_2_O_2_ solution after soaking on H BGs for 30 (a,b), 60 (c), and 120 (d,e) min.

**Figure 3:**
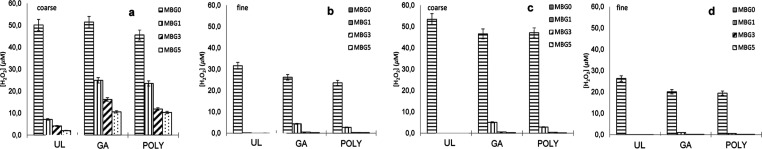
Residual H_2_O_2_ concentration (μM) of a 50 μM H_2_O_2_ solution after soaking on MBGs for 30 (a,b) and 120 (c,d) min.

**Figure 4:**
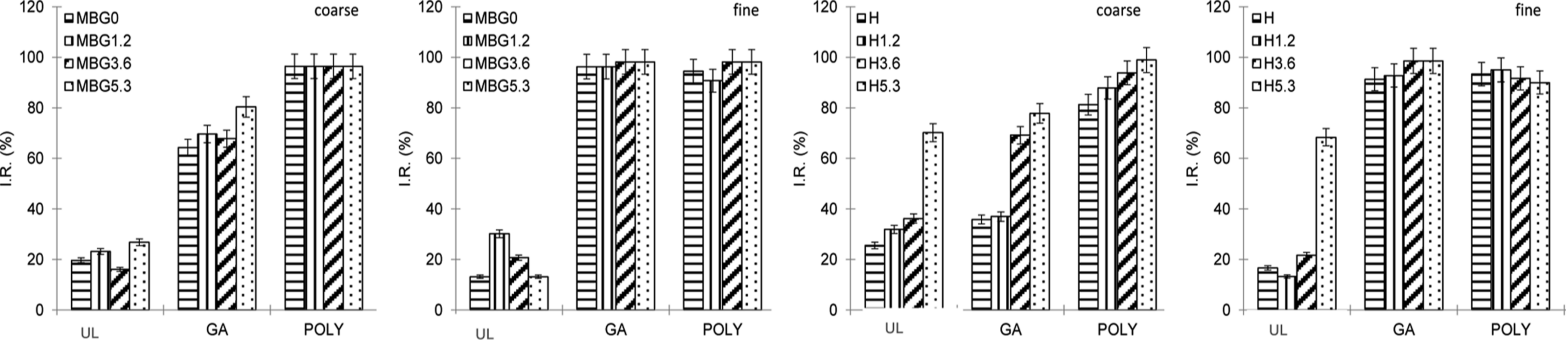
SOD-like activity of MBG and H BGs unloaded (UL) and loaded with GA and POLY, I.R. (%).

**Figure 5:**
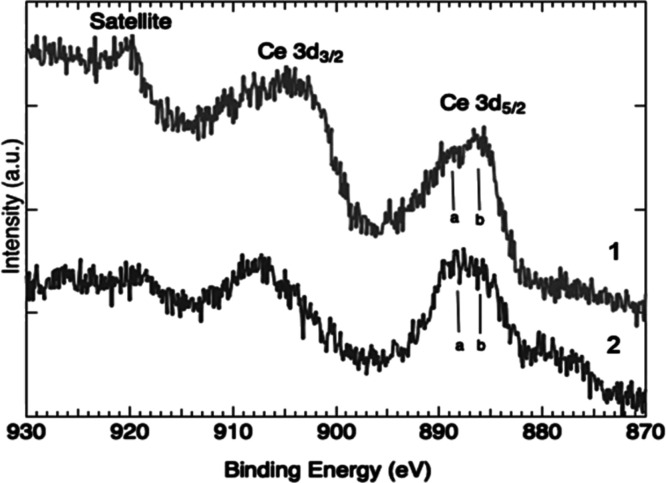
Ce 3d XPS spectra taken from MBG5.3 (1) and MBG5.3 POLY (2). The bars labeled a and b indicate the position of Ce^3+^ and Ce^4+^.

**Figure 6:**
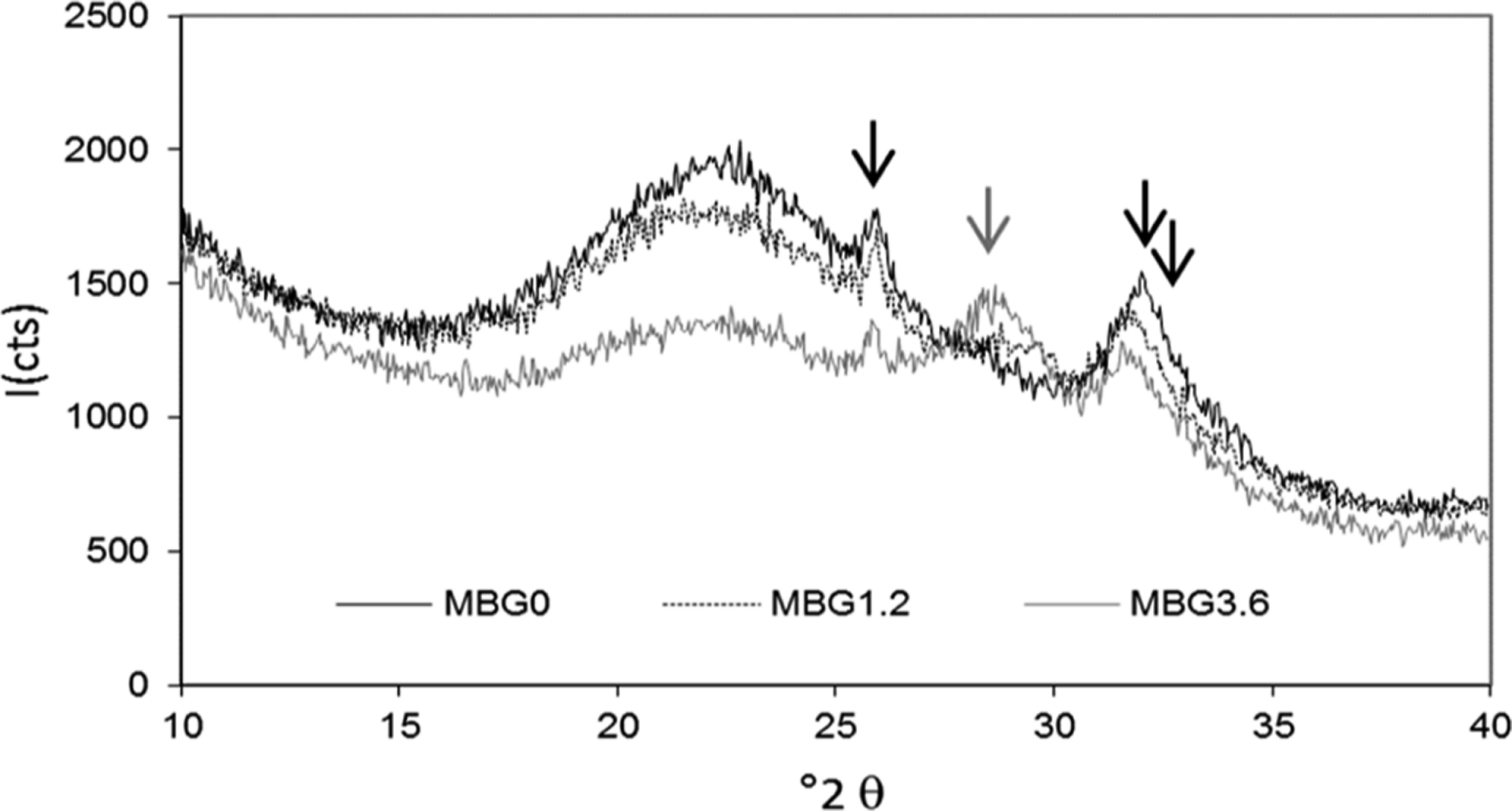
XRD powder patterns of POLY-MBGs after 168 h of soaking in SBF.

We estimated the level of dissolution of BGs upon loading by measuring the concentration of the most relevant ions in the loading solution after 3 h. Table 3 reports the release of silicon, calcium, and cerium as % of the total amount in the BG and as a function of loading solution and sizes. The corresponding histograms for silicon and calcium are reported as the Supporting Information (Figures 1–6). Calcium release in the loading solutions is roughly comparable for H and K BGs and is closely related to the sizes; dissolution for the fine size ranges between 1.0 and 2.7% and for coarse size between 0.8 and 1.6%. Lower dissolution was observed in POLY and ANTO. Possible outliers are K3.6 and K5.3BGs, which appear to show a higher dissolution rate than the other BG compositions studied, with up to 2.9% calcium dissolved in the loading solution.

**Table 3:** Release % in GA, POLY, and ANTO Solutions for Silicon, Calcium, and Cerium

	**GA**		**POLY**		**ANTO**		
**BG**	**fine**	**coarse**	**fine**	**coarse**	**fine**	**coarse**	**element**
**H0**	2.3	0.5	2.6	0.8	1.3	0.9	**silicon**
**H1.2**	1.5	0.7	2.3	0.5	N.A.^a^	N.A.^a^	
**H3.6**	2.1	0.5	2.3	0.7	N.A.^a^	N.A.^a^	
**H5.3**	2.1	0.6	2.5	0.8	1.0	0.7	
**K0**	2.0	0.6	2.1	0.8	1.0	0.7	
**K1.2**	1.9	0.5	2.0	0.5	0.9	0.5	
**K3.6**	1.6	0.5	1.0	0.5	N.A.^a^	N.A.^a^	
**K5.3**	2.4	0.5	2.5	0.4	N.A.^a^	N.A.^a^	
**MBG**	1.2	1.0	1.0	0.8	1.2	1.1	
**MBG1.2**	1.1	1.0	1.1	1.4	1.1	1.0	
**MBG3.6**	1.3	1.2	1.2	1.2	1.1	1.0	
**MBG5.3**	1.4	1.2	1.5	1.4	1.4	1.1	
**H0**	2.2	0.8	1.6	0.5	1.5	1.4	**calcium**
**H1.2**	2.2	0.7	1.5	0.6	N.A.^a^	N.A.^a^	
**H3.6**	2.3	0.8	1.7	0.5	N.A.^a^	N.A.^a^	
**H5.3**	2.3	0.6	1.8	0.6	1.3	1.0	
**K0**	2.4	1.6	1.7	2.3	1.2	1.3	
**K1.2**	2.3	1.6	1.5	1.2	1.2	1.2	
**K3.6**	2.4	1.5	1.0	2.9	N.A.^a^	N.A.^a^	
**K5.3**	2.7	1.6	2.8	2.4	N.A.^a^	N.A.^a^	
**MBG0**	8.3	7.5	4.8	4.9	4.0	3.1	
**MBG1.2**	7.9	7.1	5.0	5.1	4.3	3.8	
**MBG3.6**	6.7	6.2	4.6	3.9	4.4	4.1	
**MBG5.3**	7.0	6.4	5.0	4.8	4.0	4.5	
**H0**							**cerium**
**H1.2**	0.5	0.1	0.4	0.3	N.A.^a^	N.A.^a^	
**H3.6**	0.3	0.0	0.1	0.0	N.A.^a^	N.A.^a^	
**H5.3**	0.3	0.0	0.1	0.0	0,1	0.1	
**K0**							
**K1.2**	0.0	0.0	0.1	0.1	0.0	0.0	
**K3.6**	0.0	0.0	0.0	0.0	N.A.^a^	N.A.^a^	
**K5.3**	0.0	0.0	0.0	0.0	N.A.^a^	N.A.^a^	
**MBG0**							
**MBG1.2**	0.0	4.6	2.6	2.9	0.2	0.2	
**MBG3.6**	1.2	1.6	0.6	0.4	0.3	0.1	
**MBG 5.3**	0.5	0.3	0.6	0.1	0.2	0.1	

a Not assessed (N.A.).

Silicon dissolution for the H and K BGs is roughly in line with that of calcium, with values ranging between 0.9 and 2.6% for the fine size and a marked reduction for the coarse size BGs, which release between 0.5 and 0.9% of total silicon.

In the case of MBGs, calcium release is higher than that of H and K BGs, while silicon release is quite similar. In all cases, no particular differences are observed between the two sizes. Similarly, a slower dissolution was observed in POLY and ANTO.

These results agree with the previous literature and are explained by the higher reactivity of MBGs compared to melt-quench BGs, linked to their mesoporous texture and larger surface area.^6,56^

Cerium in general is released from all BGs only to a low extent, never exceeding 1.6% of the total cerium content, indicating that in all cases the ionic dissolution is not affected by the amount of cerium present in the BG. Again, possible outliers are present: some MBG1.2 appear to release up to 5% cerium, which is significantly different from the release trend.

### 3.3 Antioxidant Properties

#### 3.3.1 CAT-like Activity

To evaluate the CAT-like activity in a biological setting, we suspended 40 mg of BG in 400 μL of 50 μM solution of H_2_O_2_ in SBF and measured the residual concentration of H_2_O_2_ by the Amplex assay after 30, 60, and 120 min of soaking. The results are reported in Figures 2 and 3. As to be expected, cerium-free BGs without loading do not show any significant CAT-like activity, while all cerium-doped BGs are able to catalyze the dismutation of H_2_O_2_ to a relevant extent. CAT-like activity of H BGs is clearly correlated with the amount of cerium in the glass composition: coarse size of unloaded H1.2, H3.6, and H5.3 BGs reduced the H_2_O_2_ content to 5, 12, and 9 μM after 30 min and 7, 4, and 3 μM after 60 min, respectively (Figure 2a,c). All Ce-BGs show higher CAT-like activity at a fine size, with residual H_2_O_2_ concentrations of 2, <1, and 1 μM after 30 min for unloaded H1.2, H3.6, and H5.3 BGs (Figure 2b) and <1 μM after 120 min (Figure 2e).

Interestingly, loading with biomolecules seems to hamper the CAT-like activity of Ce-BGs, likely as a consequence of a reduction of the active surface available. After 30 min, both GA and POLY Ce-BGs show higher residual H_2_O_2_ compared to unloaded H1.2, H3.6, and H5.3 BGs of coarse size (30, 28, and 23 μM and 19, 16, and 7, respectively) (Figure 2a). This negative effect on CAT activity seems not only less pronounced but also less lasting for POLY loading (Figure 2c,d). Coarse H1.2, H3.6, and H5.3 POLY BGs have residual H_2_O_2_ at 4, 3, and 3 μM at 60 min and at 120 min, <1 μM for all BGs, in line with those of the unloaded BGs. Conversely, GA loading shows lasting inhibition of CAT-like activity, with 26, 21, and 9 and 15, 8, and 4 μM of residual H_2_O_2_ at 60 and 120 min, respectively.

We also tested ANTO for H and H5.3 BGs at the 30, 60, and 120 min; H5.3 ANTO BG have a behavior similar to that of H5.3 POLY, with a relatively modest inhibition of CAT activity at 60 min(8 μM), and with <1 μM of residual H_2_O_2_ after 120 min(Figure 2a,c,e). The same effects, albeit less pronounced, can be observed for H BGs at the fine particle size (Figure 2b,d). H BGs loaded with GA seem to have lower CAT activity than POLY and ANTO: for H1.2, H3.6, and H5.3, the concentrations of residual H_2_O_2_ are 7, 9, and 7 μM for GA against 7, 3, and 1 μM for POLY and 5 μM for H5.3 in ANTO. At 120 min, all Ce-BGs have residual H_2_O_2_ concentration <1 μM, further showing the higher enzyme-mimetic activities of BGs of fine size.

The behavior of MBGs closely mirrors that of H BGs but with even higher CAT activity (Figure 3), as to be expected by the higher active surface of these BGs. At 30 min, residual H_2_O_2_ concentrations for coarse unloaded MBG1.2, MBG3.6, and MBG5.3, are 7, 4, and 2 μM, while GA and POLY MBGs are slightly less active (25, 16, and 11 and 24, 12, and 10 μM for GA and POLY, respectively). At 120 min, only MBG1.2 (GA) and MBG1.2 (POLY) have measurable values of remaining H_2_O_2_ (6 and 3 μM for MBG1.2 GA and POLY respectively). At fine particle sizes, Ce-MBGs are even more active: at 30 min, MBG1.2 loaded with GA and POLY have 4 and 3 μM of residual H_2_O_2_, respectively, while all other MBGs have <1 μM H_2_O_2_. At 120 min, all Ce-MBGs completely dismutate all H_2_O_2._

#### 3.3.2 SOD-like Activity

The SOD-like activity of the BGs was evaluated by measuring the formation of the formazan dye by the superoxide anion O_2_·^–^ upon incubation of 22 mg of each BG in 220 μL of a working solution, then comparing it with the corresponding blank. The results are summarized in Figure 4 and reported as a percentage of inhibitory activity (I.R. %); higher I.R. values correspond to a better scavenging ability of O_2_•^–^. On the basis of the results from GAE % and CAT, the evaluation was focused on the most promising systems, that is, the MBGs loaded with GA and POLY. For comparison purposes, the study was also conducted on H BGs.

Intriguingly, all unloaded MBGs do not show any relevant SOD-like activity independently from the amount of cerium and size (Figure 4), with a marginal 16–30 I.R. % presumably linked to pH alteration in the medium, caused by the presence of BGs. Pleasingly, the antioxidant biomolecules confer significant SOD-like properties to the materials: for coarse sizes (Figure 4a) the loading with POLY leads to complete scavenging of the superoxide ions for all MBGs (I.R. 96%) and lowers, but still significant, I.R. values of ∼70% with GA. At fine sizes (Figure 4b), consistent with a higher loading extent of the materials linked to a higher surface area, the SOD-like activity further increases for loaded MBGs, with complete scavenging of the superoxide ion for both POLY and GA.

Distinct from MBGs, unloaded H BGs show some SOD-like activity. For an unloaded coarse size (Figure 4c), the increase of cerium amount increases SOD activity up to I.R. 70% (H5.3). A similar effect can be observed also for the loaded glasses; I.R. % of GA-loaded BGs increases from ∼40% (H) to 78% (H5.3) and with POLY, a consistent high SOD-like activity (I.R. 90–99%) was observed.

ANTO (data not shown) were assessed only for H and H5.3 BGs. They have a trend similar to that of GA, with a significant increase of SOD-like activity associated with the increase of cerium content in the glass from H (I.R. 16%) to H5.3 (I.R. 80%).

H BGs without loading and at fine sizes (Figure 4d) have values generally analogue to those of the coarse size, with an I.R. of 17 and 68% for H and H5.3 BGs, respectively. Consistent with the higher loading extent associated with fine sizes, SOD-like activity is strongly affected by the presence of antioxidant biomolecules, for example, I.R. for H5.3 increases from 68 to 99 (GA) and 90% (POLY).

#### 3.3.3 Further Characterizations

Further characterizations (SSA, FTIR, and XPS) were carried out on the most promising MBG POLY series to investigate their behavior and the loading of the biomolecules. Table 4 reports the values of the SSA before and after loading, for simplicity, only for the fine size, because no significant differences are observed for the coarse size. Consistently with the loading of biomolecules within the MBG pores, loading reduces the SSA values of all treated samples.

**Table 4:** SSA of MBGs before and after Loading with POLY

	**SSA unloaded (m^2^/g)**	**SSA loaded (m^2^/g)**
**MBG0**	318	175
**MBG1.2**	317	165
**MBG3.6**	323	190
**MBG5.3**	354	181

The FTIR spectra of loaded MBGs reported in Figure S7 (see the Supporting Information) evidence some characteristic bands of GA ,as reported in the literature,^57^ within the limited sensitivity of the technique.

The appearance of a band at 1700 cm^–1^ representing the C=O axial deformation, one around 1500 cm^–1^ representing the C=C axial deformation in aromatics, and one band at 1400 cm^–1^ indicated the O–H deformation indicate the presence of GA in the MBGs. Other bands, such as the one near 3200 cm^–1^ due to the O–H axial deformation, at 3100 cm^–1^ due to the aromatic C–H axial deformation, the C–O axial deformation at 1250 cm^–1^, and the C–H axial deformation in aromatics at 650 cm^–1^ are not always visible, probably because they are hidden by the IR profile of MBGs. In all cases, doping with cerium does not alter the loading behavior and the GA signals are visible in all treated samples.

XPS spectra from MBG 5.3 and MBG 5.3 POLY are shown in Figure 5. In agreement with our previous studies,^17−20,22^ on the surface of the MBGs, both oxidation states of cerium are present. The low signal hampers quantitative analysis of the relative concentration between the Ce^4+^ and Ce^3+^ ionic species. On the other hand, the main structure, corresponding to emission from Ce 3d_5/2_, shows a significant change.

The addition of a reducing species (POLY) slightly alters the ratio between the two oxidation states without inhibiting the antioxidant properties, as shown by the interesting results of the tests described above. Further evidence of reduction is the substantial decrease of the satellite, typical of spectra of nanoceria.

### 3.4 *In vitro* Bioactivity Assessment

In order to confirm the ability of the loaded BGs to maintain their bioactivity in a biological medium, we carried out a mineralogical evaluation to verify the presence of HA. MBGs loaded with GA and POLY were soaked for 24, 96, and 168 h in SBF. In all cases, we can affirm that these BGs are still bioactive. For clarity, we report the patterns obtained after 168 h (Figure 6) only for three POLY-MBGs; the results after 24 and 96 h and for MBG5.3 are not shown simply because the peaks are less defined but belong to those of HA. Figure 6 highlights the formation of an apatite layer; peaks at 26, 31, and 33 (°2θ) (indicated with black arrows) belong to those of HA.^55^ MBG3.6 also shows a peak, indicated with gray arrows, at ∼ 29 (°2θ) that belongs to CePO_4_, a phosphate competitive with HA formation, slowing its formation, as reported previously.^46,58^

Evaluation of the release of constituent ions was performed after 24 and 168 h of soaking in SBF. The results are shown in Table 5 and agree with previous studies on unloaded MBGs, showing the dissolution of MBGs to form an apatite layer.^59^ Ions’ release is thus not influenced by loading with POLY. Dissolution is slightly slowed by cerium ions, and the amount of cerium released is always less than 1 μg/L.

**Table 5:** Ions Leaching (mM) in SBF for POLY-MBGs

	**calcium**		**silicon**		**phosphorus**	
	**24 h**	**168 h**	**24 h**	**168 h**	**24 h**	**168 h**
**MBG0**	2.7	2.4	2.0	3.5	0.2	0.1
**MBG1.2**	2.5	2.4	2.0	3.6	0.2	0.1
**MBG3.6**	2.5	2.3	1.9	2.9	0.3	0.2
**MBG5.3**	2.7	2.6	1.7	2.6	0.2	0.2

## 4 Conclusions

We evaluated the properties of different cerium-doped BGs: melt-quenched H and K series and sol–gel-derived MBG series.

BGs were loaded with antioxidant biomolecules both pure (GA) and as mixtures extracted from natural products (POLY and ANTO).

Quantification of loading at variable times highlighted a range of contents dependent on both the BG and biomolecule types, with the best results observed for the loading of POLY on MBGs.

A soaking time of 3 h allowed one to reach the equilibrium between the BGs and the loading solution, limiting the dissolution of the BGs measured as a loss of calcium, silicon, and cerium.

Evaluation of the antioxidant properties highlighted a pattern in which the ability to dismutate hydrogen peroxide (CAT-like activity) is strongly correlated with cerium doping, while it shows a marginal decrease compared to the parent BG upon loading with biomolecules.

Conversely, unloaded cerium-doped BGs show little (H, K) or no (MBG) ability to dismutate the superoxide anion (SOD-like activity), but loading with biomolecules, especially POLY, greatly enhances the SOD-like ability of the final materials.

Doping with cerium and loading with biomolecules also add complementary antioxidant properties to the BGs; combined with the persistent bioactivity, this makes these materials prime candidates for upcoming studies on biological systems.

## Supporting Information Available

Loading of biomolecules; silicon and calcium concentrations (mg/L) of H and K BGs; and FTIR spectra (PDF)
