# Supplementary material for: Loading with Biomolecules Modulates the Antioxidant Activity of Cerium-Doped Bioactive Glasses
Source: ACS Biomater Sci Eng. 2022 Jun 13;8(7):2890–8. doi: 10.1021/acsbiomaterials.2c00283 (PMC9937534; doi:10.1021/acsbiomaterials.2c00283)
Supplement: Supplementary file 1 — ab2c00283_si_002.pdf [file ab2c00283_si_002.pdf]

Supplementary Information pertaining to:

Loading with Biomolecules modulates the antioxidant activity of cerium-doped Bioactive Glasses

Gigliola Lusvardi,[a]* Francesca Fraulini,[a] Sergio D'Addato,[b,c] Alfonso Zambon[a]*

[a]Department of Chemical and Geological Sciences, University of Modena and Reggio Emilia, Via G.Campi 103, 41125, Modena, Italy

[b]Department of Physical, Information and Mathematical Sciences, University of Modena and Reggio Emilia, Via G. Campi 213/a, 41125, Modena, Italy

[c]Istituto Nanoscienze−CNR, Via G. Campi 213/a, 41125 Modena, Italy

** corresponding authors: gigliola.lusvardi@unimore.it, alfonso.zambon@unimore.it*

Table S1 Loading of biomolecules, expressed as GAE% on H,K BGs as obtained from F&C and EA.
S2

Figure S1a,b,c Silicon concentration (mg/L) of H BGs after soaking in GA (a), POLY(b), ANTO (c) solutions.
S3

Figure S2 a,b,c Silicon concentration (mg/L) of K series after soaking in GA (a), POLY(b), ANTO (c) solutions.
S4

Figure S3a,b,c Silicon concentration (mg/L) of MBG series after soaking in GA (a), POLY(b), ANTO (c) solutions.
S5

Figure S4,a,b,c Calcium concentration (mg/L) of H series after soaking in GA (a), POLY(b), ANTO (c) solutions.
S6

Figure S5a,b,c Calcium concentration (mg/L) of K series after soaking in GA (a), POLY(b), ANTO (c) solutions.
S7

Figure S6,a,b,c Calcium concentration (mg/L) of MBG series after soaking in GA (a), POLY(b), ANTO (c) solutions.
S8

Figure S7 FTIR spectra of MBGs unloaded (black) and loaded with POLY (light grey).
S9-S12

Table S1 Loading of biomolecules, expressed as GAE%, on H,K BGs as obtained from F&C and EA

		GA				POLY				ANTO	
		F&C		EA		F&C		EA		F&C	
		3h	6h	3h	6h	3h	6h	3h	6h	3h	6h
coarse	H0	0.01	0.05	0.12	0.18	0.18	0.14	0.28	0.18	0.04	0.04
	H1.2	0.02	0.05	0.14	0.12	0.14	0.12	0.34	0.38	N.A.*	N.A*.
	H3.6	0.03	0.07	0.02	0.08	0.19	0.14	0.36	0.26	N.A.*	N.A*.
	H5.3	0.02	0.03	0.20	0.10	0.24	0.14	0.38	0.40	0.01	0.03
fine	H0	0.17	0.12	0.10	0.06	0.35	0.05	0.32	0.02	0.07	0.09
	H1.2	0.06	0.04	0.14	0.16	0.22	0.09	0.26	0.24	N.A.*	N.A*.
	H3.6	0.12	0.04	0.02	0.04	0.27	0.12	0.34	0.22	N.A.*	N.A*.
	H5.3	0.20	0.16	0.18	0.14	0.15	0.29	0.08	0.34	0.05	0.06
coarse	K0	0.01	0.02	N.A*.	N.A*.	0.24	0.25	0.26	N.A*.	0.03	0.03
	K1.2	0.01	0.04	0.24	N.A*.	0.17	0.16	0.39	N.A*.	N.A*.	N.A*.
	K3.6	0.01	0.02	N.A.*	N.A*.	0.20	0.14	0.34	N.A*.	N.A*.	N.A*.
	K5.3	0.01	0.02	N.A.*	N.A*.	0.22	0.17	0.34	N.A*.	0.04	0.03
fine	K0	0.22	0.15	0.34	N.A*.	0.36	0.21	0.44	0.18	0.05	0.06
	K1.2	0.19	0.08	N.A.*	N.A*.	0.27	0.39	0.30	0.40	N.A*.	N.A*.
	K3.6	0.01	0.09	N.A.*	N.A*.	0.31	0.08	0.22	0.04	N.A*.	N.A*.
	K5.3	0.01	0.06	N.A.*	N.A*.	0.17	0.03	0.12	N.A*.	0.04	0.03

* = not assessed (N.A.)

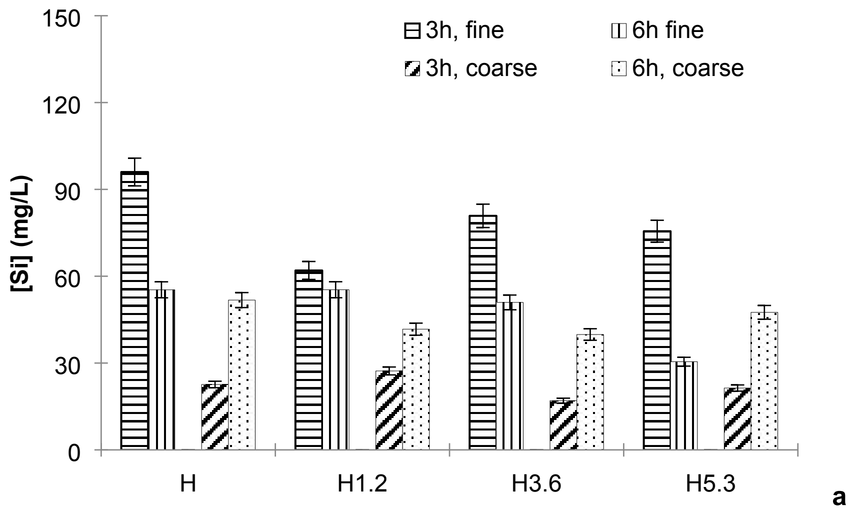

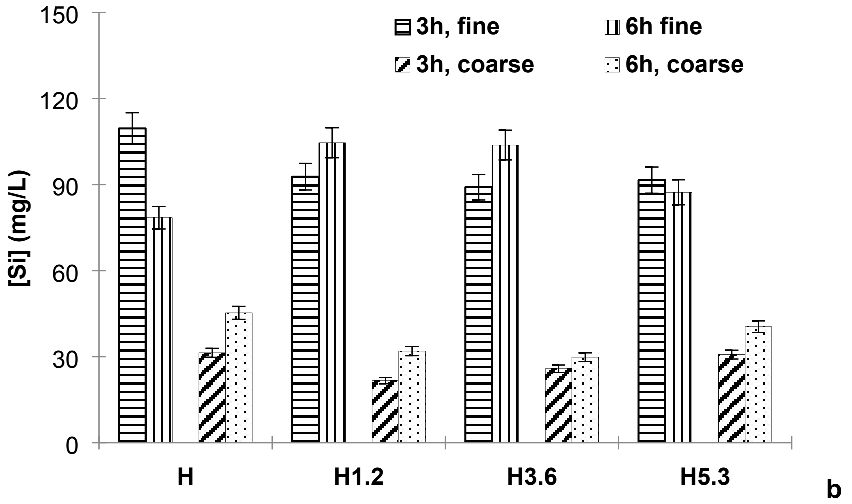

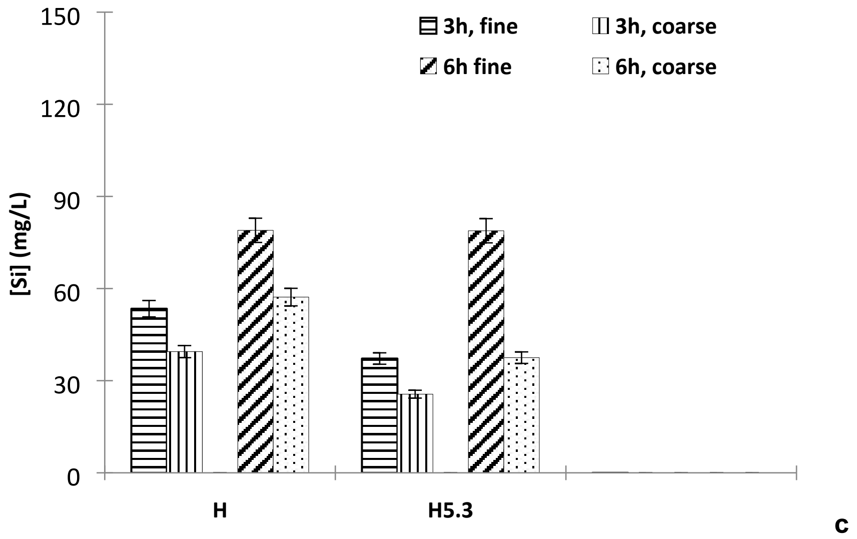

Figure S1a,b,c Silicon concentration (mg/L) of H BGs after soaking in GA (a), POLY(b), ANTO (c) solutions.

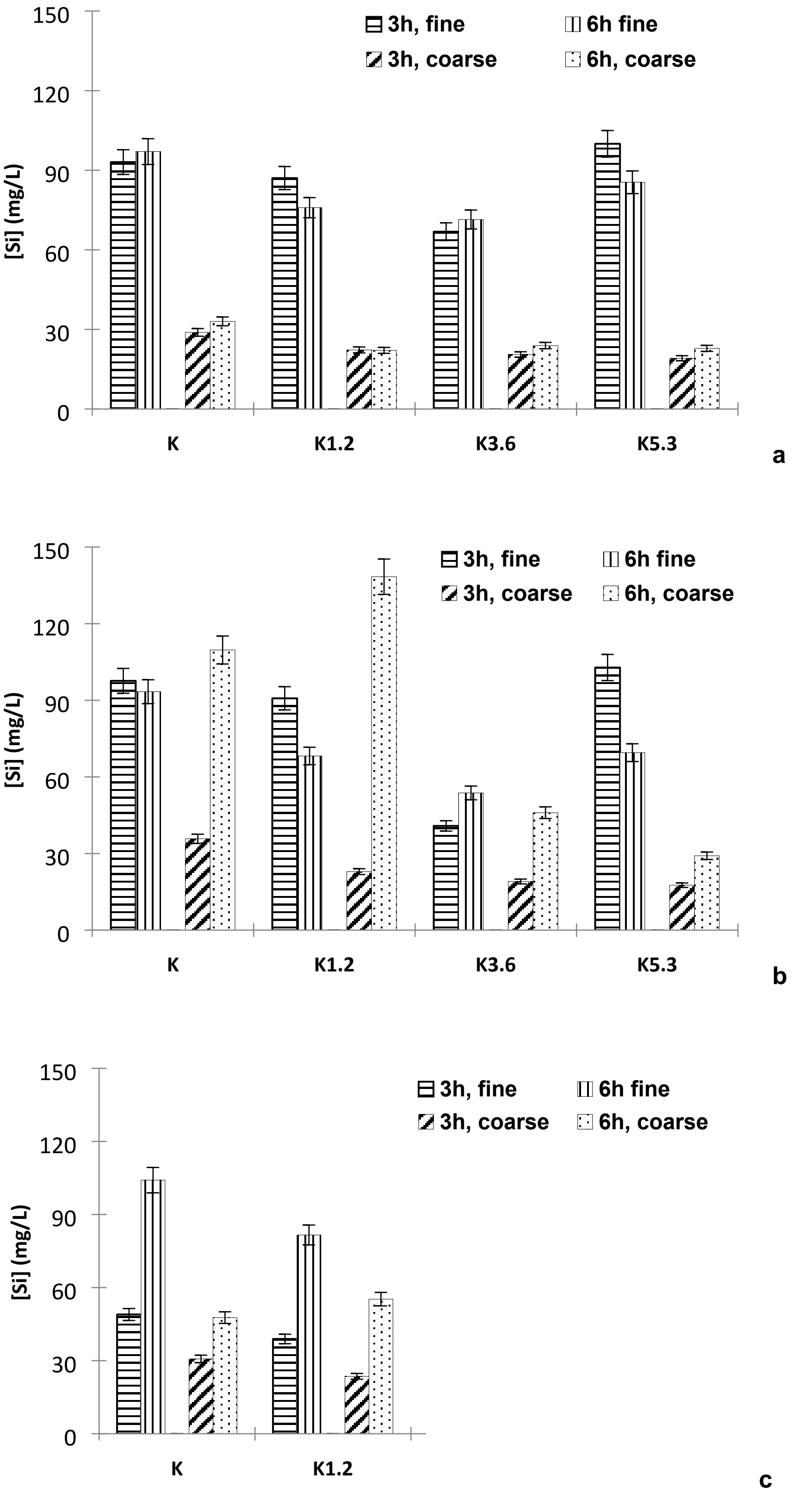

Figure S2 a,b,c Silicon concentration (mg/L) of K series after soaking in GA (a), POLY(b), ANTO (c) solutions.

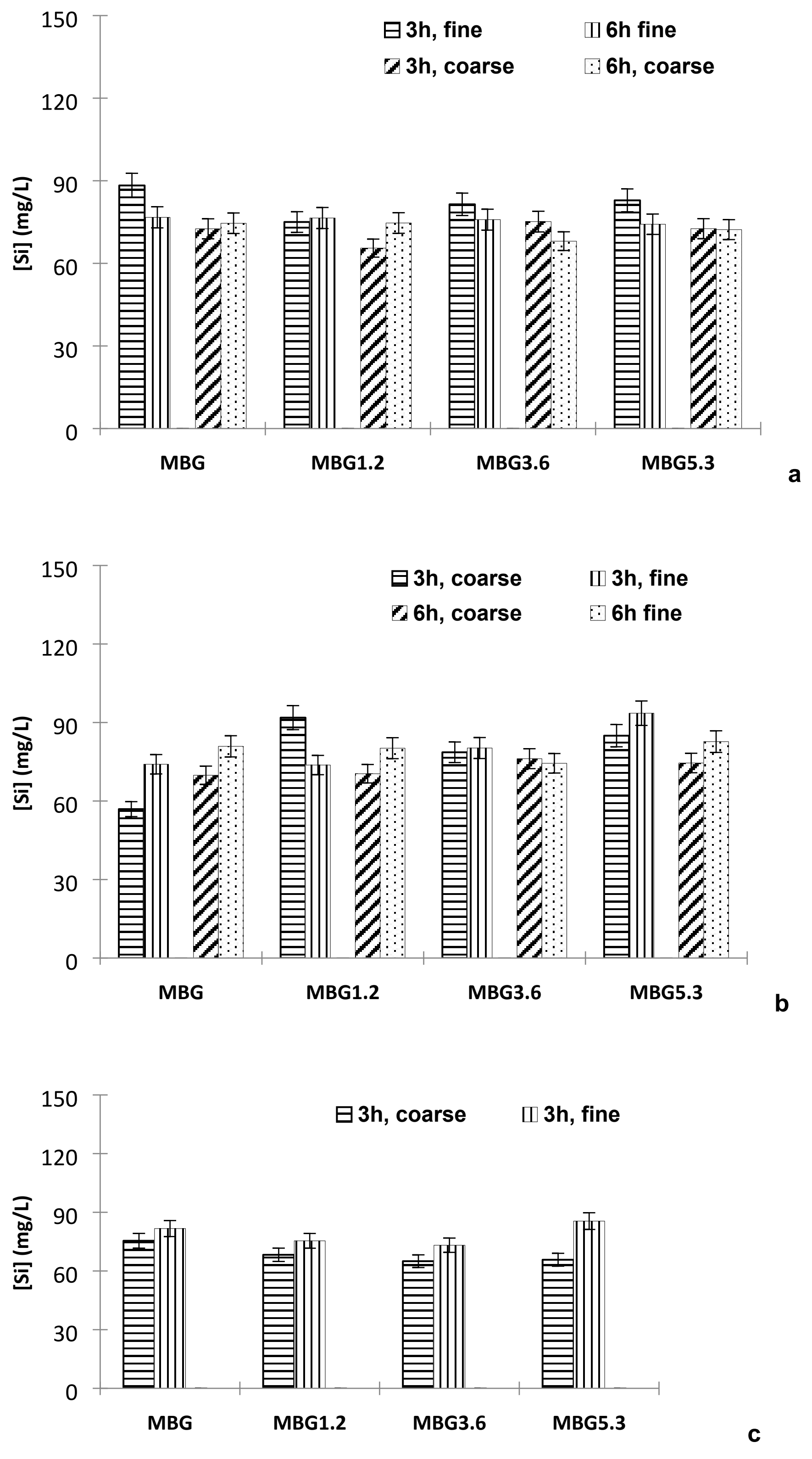

Figure S3a,b,c Silicon concentration (mg/L) of MBG series after soaking in GA (a), POLY(b), ANTO (c) solutions.

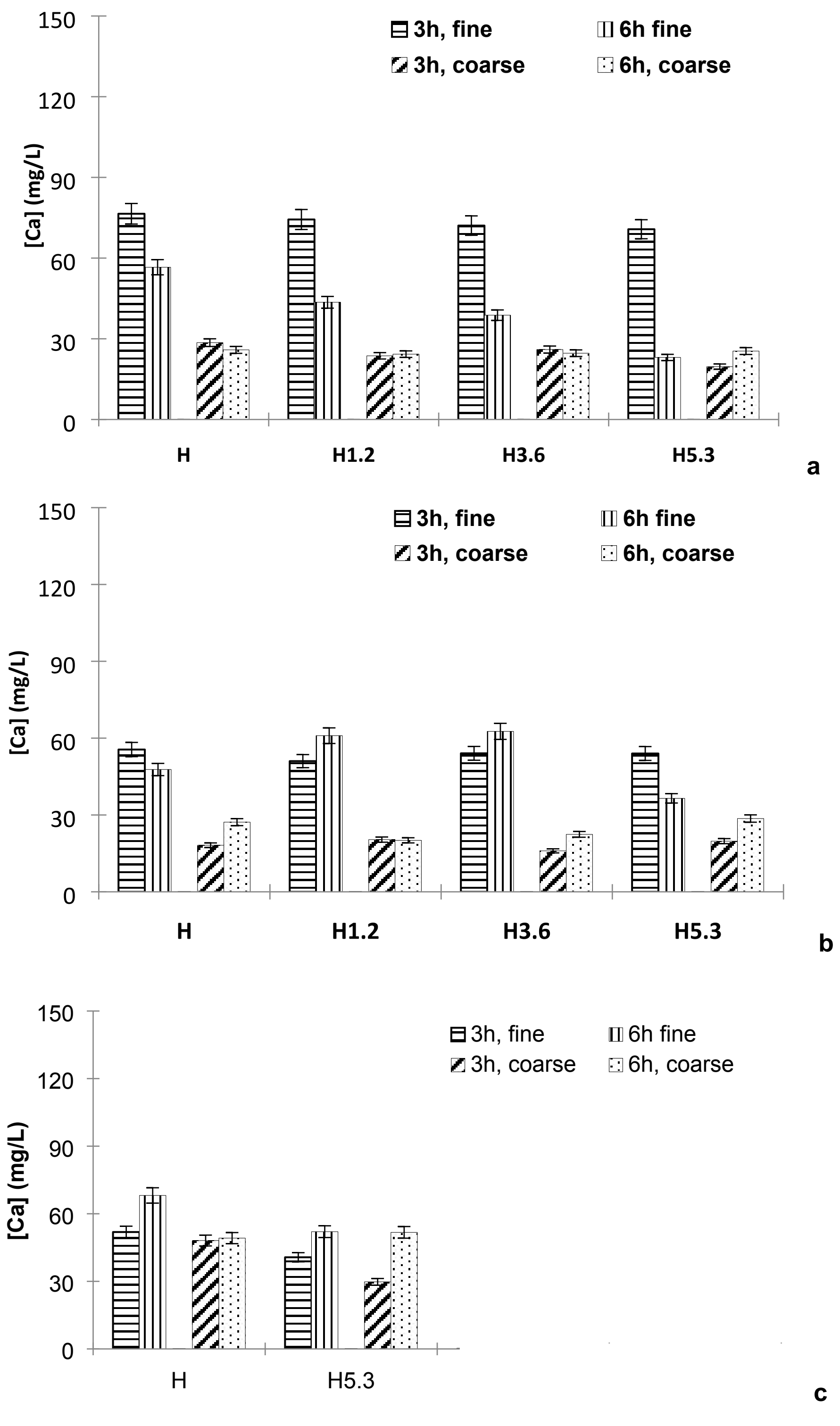

Figure S4,a,b,c Calcium concentration (mg/L) of H series after soaking in GA (a), POLY(b), ANTO (c) solutions.

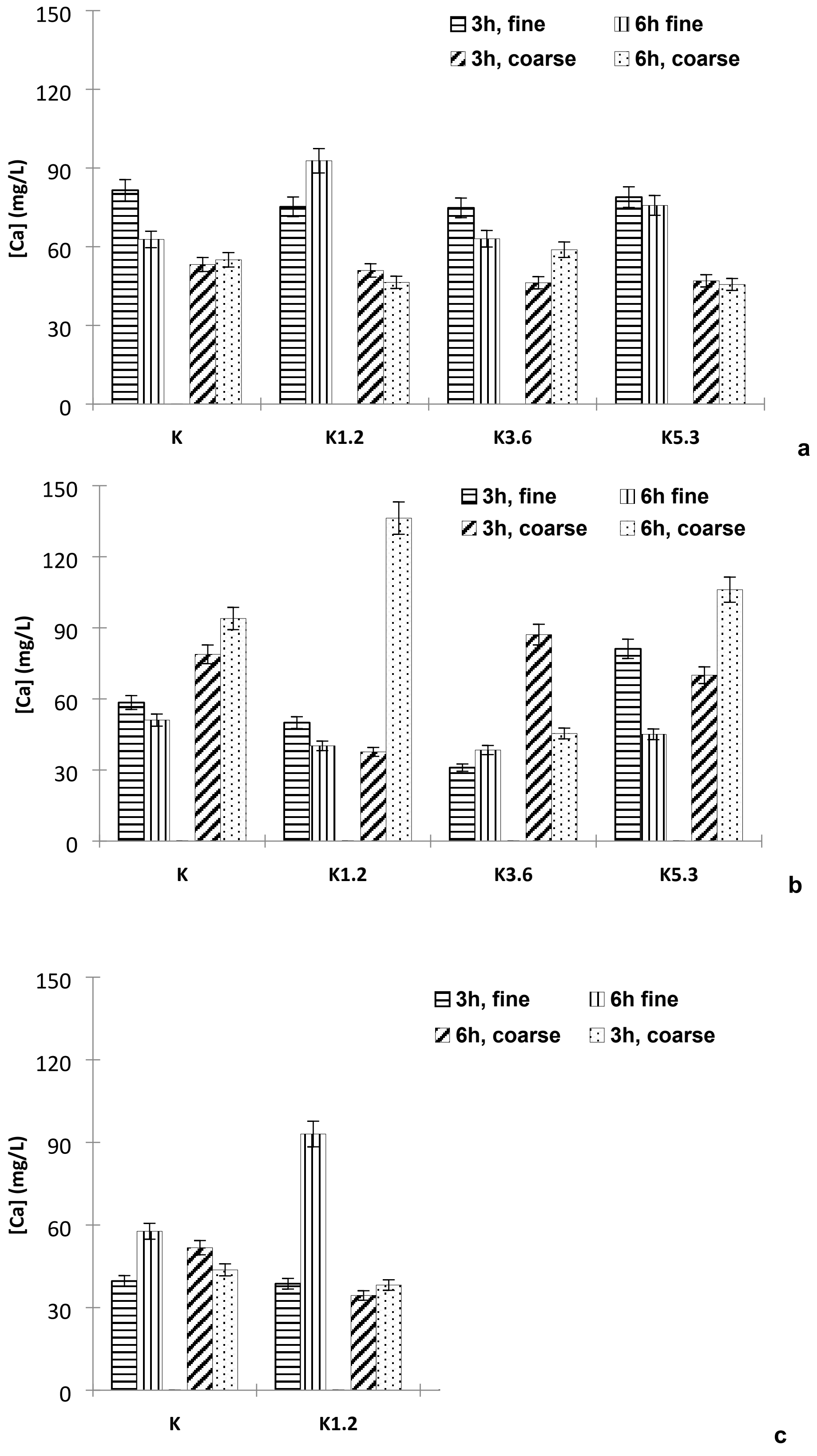

Figure S5a,b,c Calcium concentration (mg/L) of K series after soaking in GA (a), POLY(b), ANTO (c) solutions.

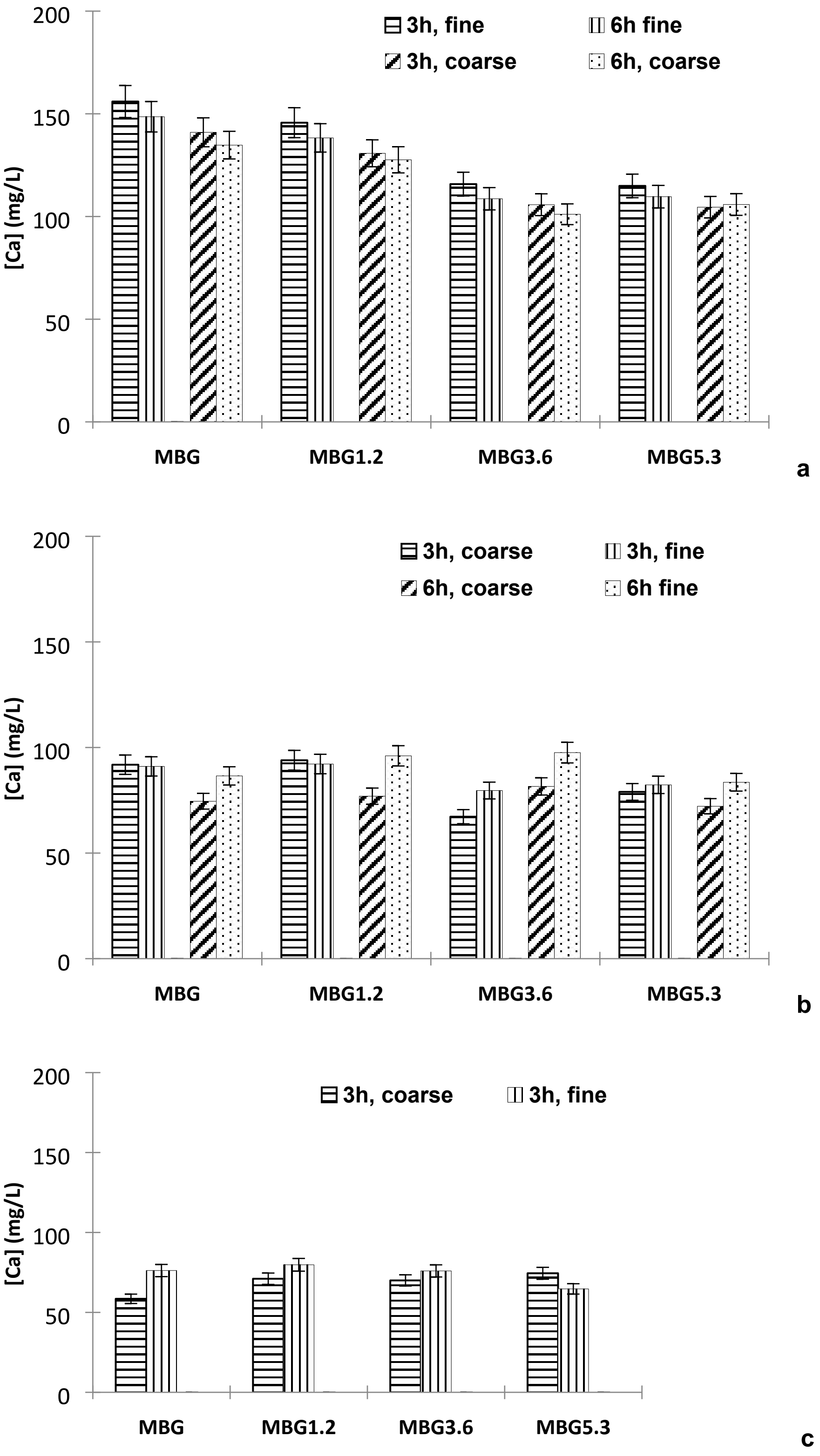

Figure S6,a,b,c Calcium concentration (mg/L) of MBG series after soaking in GA (a), POLY(b), ANTO (c) solutions.

MBG

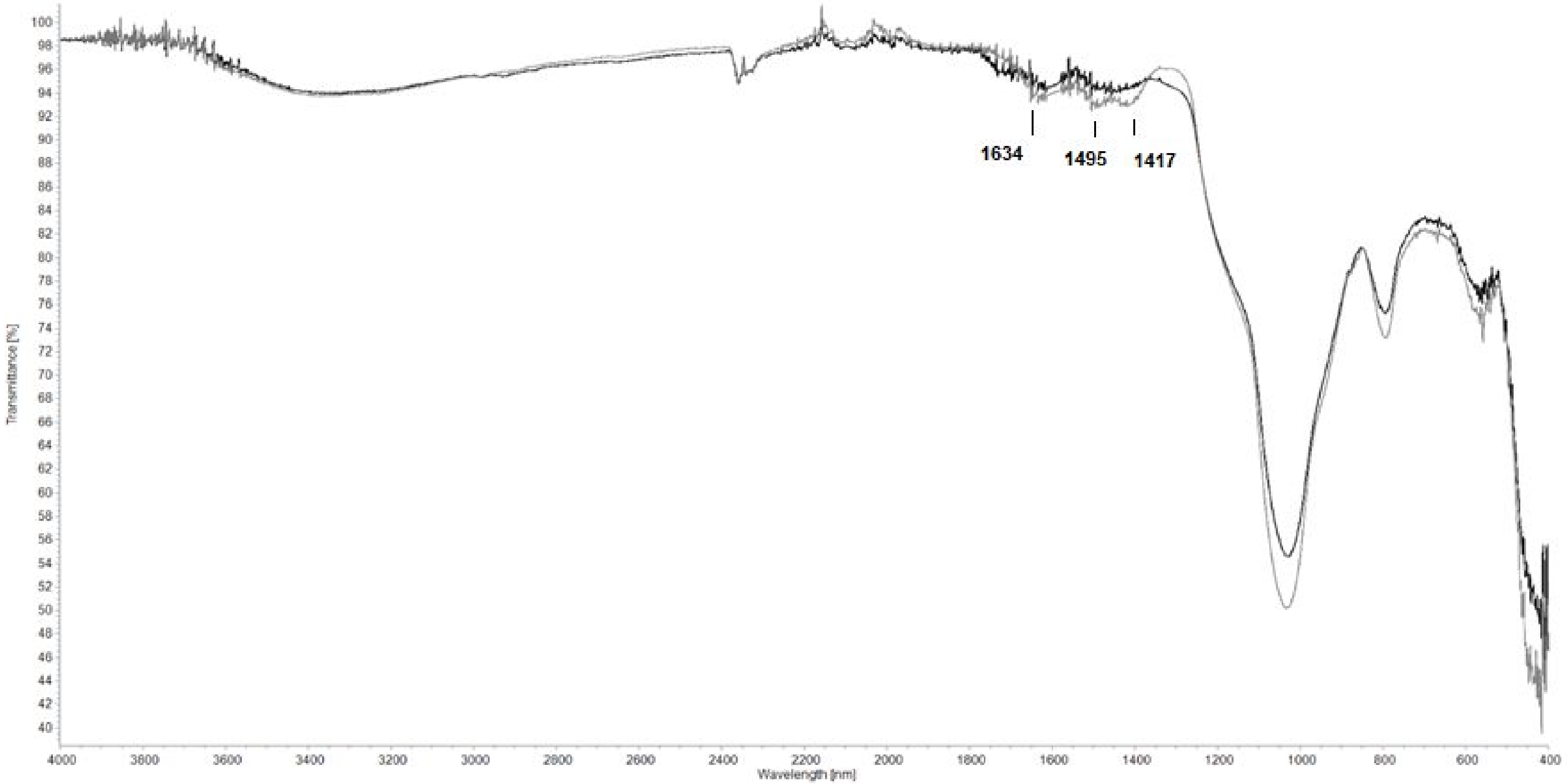

MBG 1.2

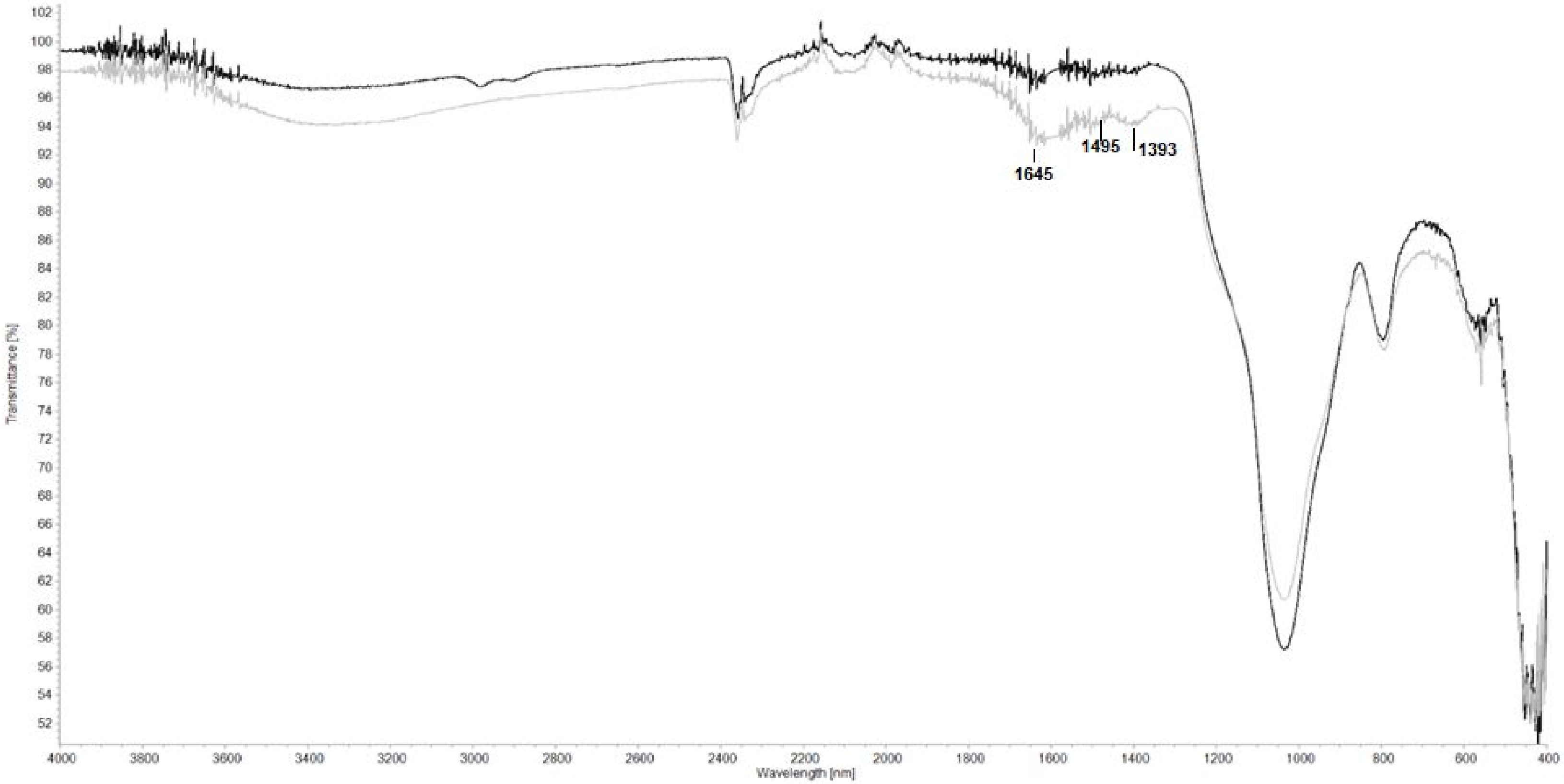

MBG 3.6

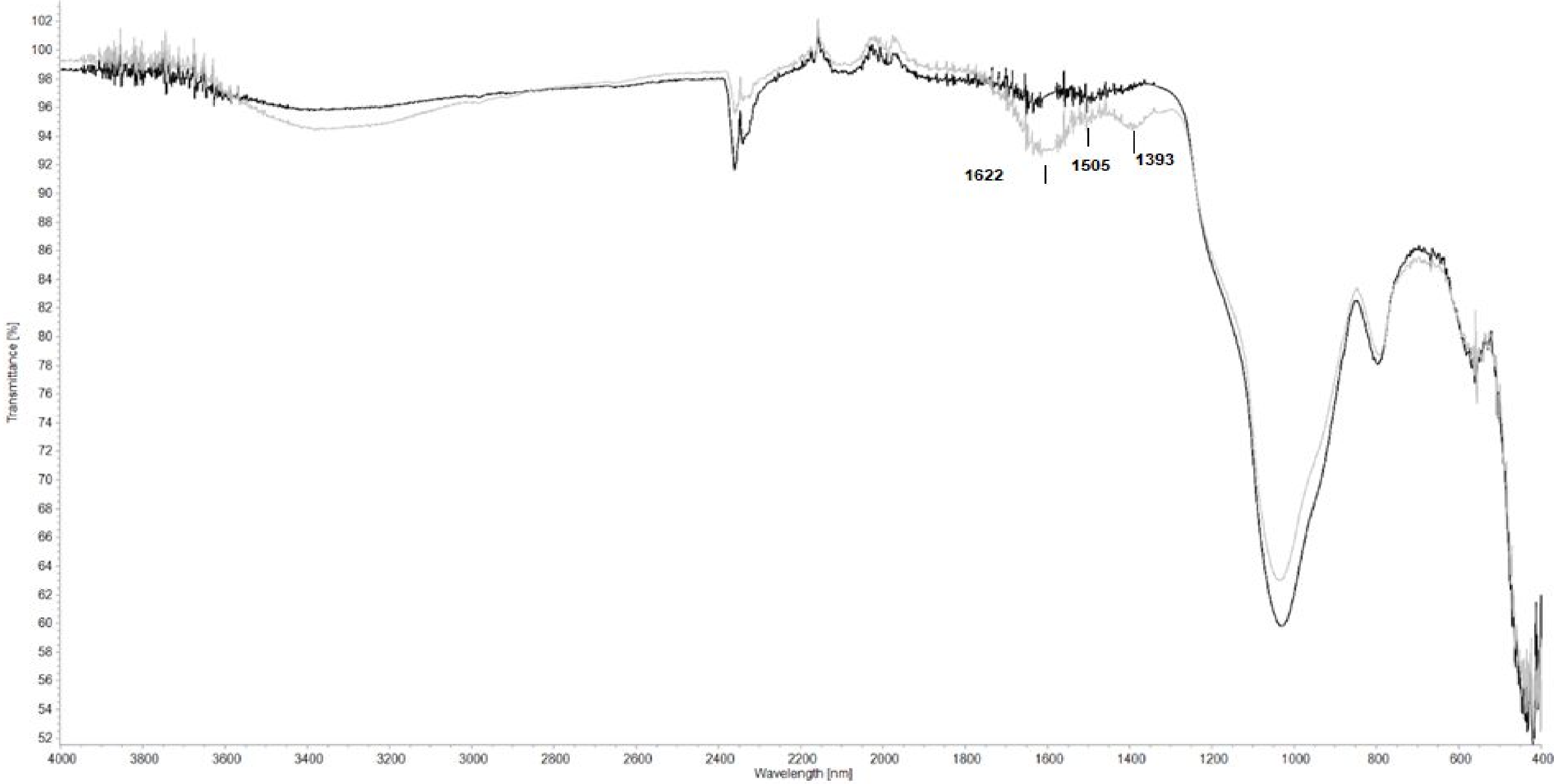

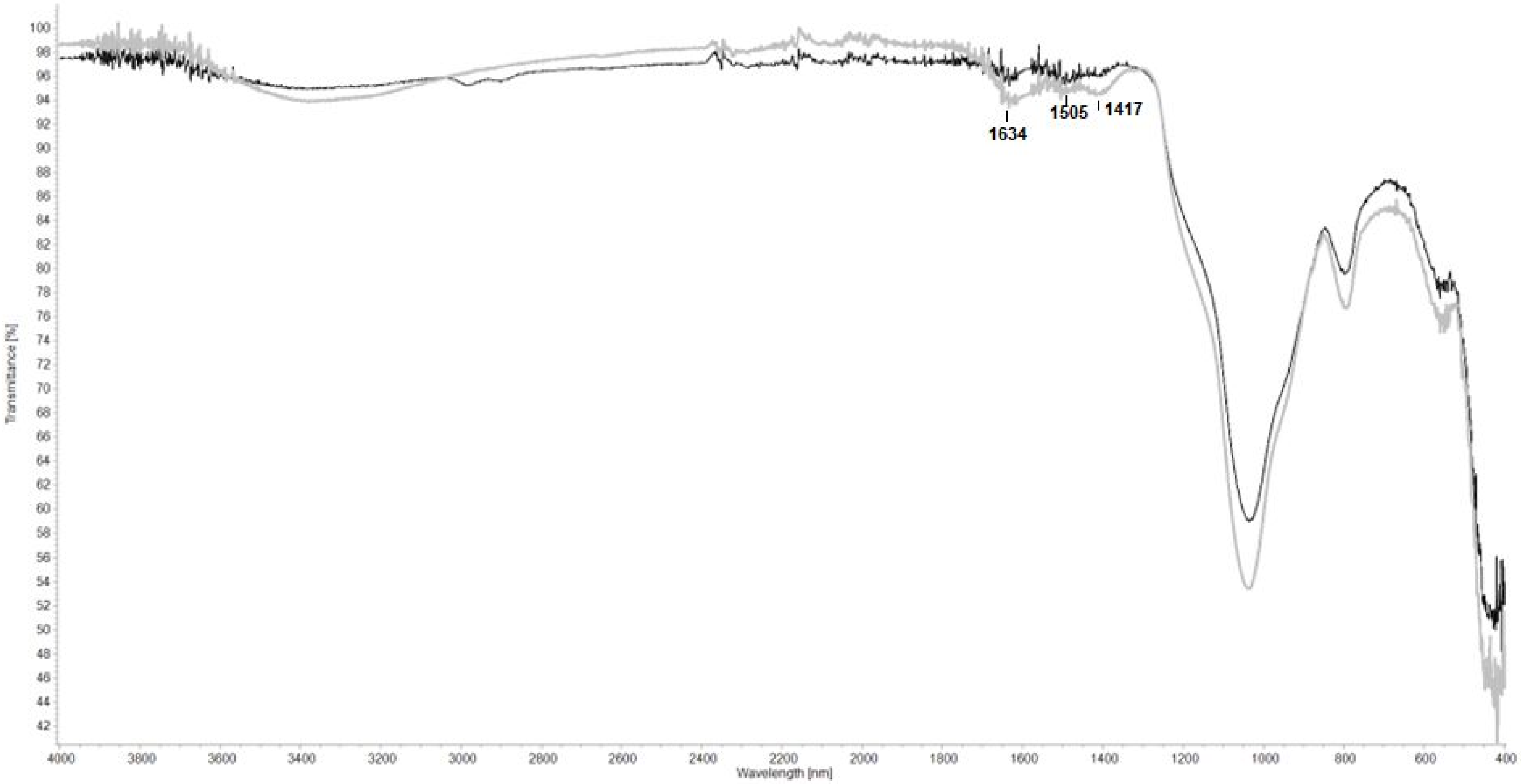

Figure S7 FTIR spectra of MBGs unloaded (black) and loaded with POLY (light grey).
